# IL-25 blockade inhibits metastasis in breast cancer

**DOI:** 10.1007/s13238-016-0345-7

**Published:** 2016-12-01

**Authors:** Zhujun Jiang, Jingtao Chen, Xuemei Du, Hang Cheng, Xiaohu Wang, Chen Dong

**Affiliations:** 1 Institute for Immunology and School of Medicine, Tsinghua University, Beijing, 100084 China; 2 Institute of Translational Medicine, The First Hospital, Jilin University, Changchun, 130031 China; 3 Department of Pathology, Beijinɡ Shijitan Hospital of Capital Medical University, Beijing, 100038 China; 4 Department of Pediatrics, The First Hospital, Jilin University, Changchun, 130031 China

**Keywords:** IL-25, breast cancer, metastasis, MMTV-PyMT

## Abstract

Metastasis is the leading cause of death in breast cancer patients. However, the mechanisms underlying metastasis are not well understood and there is no effective treatment in the clinic. Here, we demonstrate that in MMTV-PyMT, a highly malignant spontaneous breast tumor model, IL-25 (also called IL-17E) was expressed by tumor-infiltrating CD4^+^ T cells and macrophages. An IL-25 neutralization antibody, while not affecting primary tumor growth, substantially reduced lung metastasis. Inhibition of IL-25 resulted in decreased type 2 T cells and macrophages in the primary tumor microenvironments, both reported to enhance breast tumor invasion and subsequent metastasis to the lung. Taken together, our data suggest IL-25 blockade as a novel treatment for metastatic breast tumor.

**Electronic supplementary material:**

The online version of this article (doi:10.1007/s13238-016-0345-7) contains supplementary material, which is available to authorized users.

## INTRODUCTION

Breast cancer is the most common, invasive cancer in women. Although early diagnosis and surgical removal of the primary tumor can effectively reduce the mortality, tumor relapse and metastasis to lung, liver and bone marrow remain a primary cause of death in breast cancer patients. Different mouse models have been developed to study the mechanism underlying human breast cancer development. For examples, intravenous injection of tumor cells are useful in studying tumor dissemination and colonization to the secondary organ, while syngeneic and xenograft tumor transplantation models are often used to study the mechanism of tumor invasiveness at early and later stages. Genetically engineered mouse models, such as over-expression of oncogenes (i.e. c-myc (McCormack et al., 1998; D’Cruz et al., 2001), ErbB2 (Guy et al., 1992; Moody et al., 2002, PyMT (Maglione et al., 2001), Wnt1 (Tsukamoto et al., 1988), and RAS (Nielsen et al., 1991)) or disruption of tumor suppressor genes (p53 (Lin et al., 2004), Brca1 (Venkitaraman 2002), or Pten (Di Cristofano and Pandolfi 2000) for example), are particularly useful in investigating the progression of in situ breast tumor development and spontaneous metastasis.

Earlier studies based on various tumor models have revealed an essential role of immune cells in controlling metastasis of breast tumors. Among them, tumor-associated macrophages were implicated in all stages in tumor metastasis by modifying the behavior of cancer cells (Noy and Pollard, 2014; Kitamura et al., 2015). Deficiency in *Csf1*, the key gene controlling the development of macrophages, significantly reduced tumor metastasis to the lung in the MMTV-PyMT transgenic mice (Lin et al., 2001). Further studies indicate that tumor-associated macrophages promote metastasis by producing pro-angiogenic factors such as VEGF and matrix metalloproteinase (Giraudo et al., 2004; Lin et al., 2006). Moreover, tumor-associated macrophages can suppress the infiltration and anti-tumor response of CD8+ T cells and NK cells (Noy and Pollard, 2014). Tumor-associated macrophages could also directly promote invasion and extravasation of tumor cells by secreting cytokines and extracellular matrix-remodeling enzymes.

Several lines of evidences have also demonstrated an important role of Th2 cells in controlling the progress and metastasis of tumors (Coussens et al., 2013), particularly through secreting interleukin-4 (IL-4), which may modulate the polarization and effector function of type 2 macrophages (M2). For examples, IL-4 was reported to promote the growth, angiogenesis, and invasion of tumor cells by inducing cathepsin activity in macrophages (Gocheva et al., 2010). Th2 cells, through secreting IL-4, were shown to potentiate pulmonary metastasis through M2 macrophages to activate epidermal growth factor receptor (EGFR) signaling in malignant mammary epithelial cells (DeNardo et al., 2009). Interestingly, high levels of Th2 cell-derived cytokines were reported in the sera of different types of human breast cancer patients, and the levels of IL-4 and the number of tumor-infiltrating CD4+ T cells were found to positively correlate with tumor progression and metastasis to sentinel lymph nodes (Pedroza-Gonzalez et al., 2011; Kohrt et al., 2005; Mantovani et al., 2008), highlighting the clinical relevance of type 2 immune cells in the pathogenesis of human breast tumors.

IL-25 (also named as IL-17E), a member of the IL-17 family cytokines, has been shown to be expressed by many different types of cells, including lung and intestinal epithelial cells, Th2 cells, mast cells, eosinophils, basophils, macrophages etc, with its receptor IL-17RB expressed by fibroblasts, epithelial cells, Th2 cells, ILC2 cells and macrophages (Licona-Limon et al., 2013; Fahy 2015). Previous studies by others and us have demonstrated an important role of IL-25 in regulating the differentiation and function of Th2 cells and type 2 immune response in various models (Fort et al., 2001; Angkasekwinai et al., 2007; Wang et al., 2007; Barlow and McKenzie 2009).

The role of IL-25 in breast cancer has remained largely unknown. Of particular interests, recent studies suggest that IL-25 may play an anti-tumor role in several in vitro and in vivo systems. For examples, IL-25 was shown to directly act on and induce apoptosis of breast cancer cells (Furuta et al., 2011). In addition, treatment with IL-25 was reported to reduce tumor growth in several xenograft tumor models, including melanoma, breast, lung, colon, and pancreatic cancers (Benatar et al., 2010). In contrast to these observations, elevated expression of IL-25 receptor IL-17RB was shown to have a strong correlation with poor prognosis in breast cancer patients and a strong pro-tumor activity in breast tumor models, through regulating NF-κB-mediated anti-apoptotic pathway (Huang et al., 2014), or inflammatory chemokine expression, including CCL20/CXCL1/IL-8/TFF1 (Wu et al., 2015). In this work, we investigated the role of IL-25 in a widely used spontaneous breast cancer model, the MMTV-PyMT transgenic mice with lung as the major metastasis site (Guy et al., 1992). We found that IL-25 promoted tumor metastasis to the lung, possibly via regulating type 2 immune responses in the tumor microenvironments.

## RESULTS

### IL-25 expression in mammary adenocarcinoma

**Figure 1:**
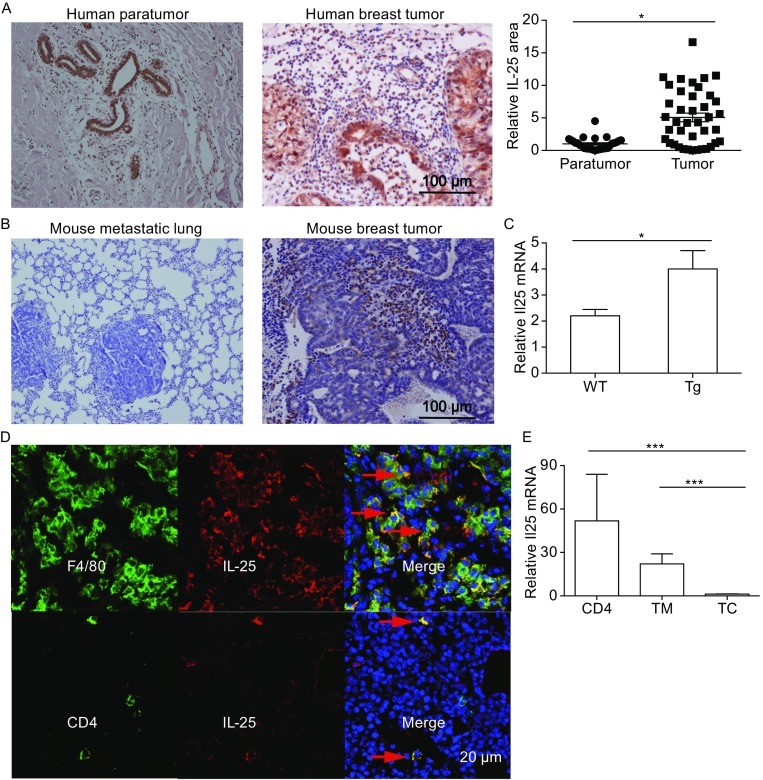
**Macrophages and CD4 cells expressed IL-25 inside mammary adenocarcinoma**. (A) Image Pro Plus characterized IHC-staining of IL-25 (×10 magnification) in human breast paratumor and carcinoma, and the IL-25+ cells through counting the relative positive area. (B) IHC-staining of IL-25 in mouse breast carcinoma and metastatic lung tissues of the MMTV-PyMT transgenic mice. (C) The relative mRNA level of Il25 in breast tissues isolated from wide type and the MMTV- PyMT mice (14 weeks old) WT: wide type mice; Tg: MMTV- PyMT transgenic mice. (D) Immunofluorescence staining of IL-25 (red), CD4 (green) or F4/80 (green) in the mammary carcinomas tissues of the MMTV-PyMT mice, and the merged images were shown at the left side. (E) The relative Il25 mRNA level in the tumor infiltrating CD4+ T cells (CD4: CD45+CD11b-CD3+CD4+), macrophages (TM: CD45+CD11b+F4/80+Gr-1-) and CD45- cells (TC) isolated from the mammary carcinomas of MMTV-PyMT mice (14 weeks old)

IL-25 has been reported to express in breast tumor specimen (Mombelli et al., 2015). For a further understanding of the clinical relevance of IL-25, we examined IL-25 expression in all the four major molecular subtypes of human breast tumors, including Luminal A, Luminal B, Triple negative/basal-like, and HER2-enriched types, by immunohistochemical analysis. To our surprise, IL-25 expression was observed in all the 40 clinical sections examined according to immunohistochemistry staining (Fig. S1A). It is noted that tumor-associated epithelial cells expressed higher levels of IL-25 compared with the para-tumor areas as shown by the positive areas (Fig. 1A). However, we did not found any difference of IL-25 expression levels among four groups through counting integrated optic density by Image Pro Plus (Fig. S1B). Interestingly, tumor-infiltrating cells also exhibited copious IL-25 expression in the tumor, in all the four types of breast cancers examined.

To further study the role of IL-25 in breast cancer development, we utilized a mouse model of spontaneous breast tumor - the MMTV-PyMT transgenic mouse. In this model, the polyoma virus middle T antigen is expressed under the control of mammary gland-specific promoter MMTV. A major advantage of this model is that all the transgenic mice will develop spontaneous multifocal mammary adenocarcinomas and lung metastasis within a relative short time period and tumor progression closely mimics human breast cancer at four distinct stages, i.e. hyperplasia (4–6 weeks old), adenoma/mammary intraepithelial neoplasia (8–9 weeks old), and early carcinoma (8–12 weeks old) and late carcinoma (over 10 weeks old) (Lin et al., 2003). Tumor microenvironments at both primary and metastatic sites are known to play an important role in metastasis (Chambers et al., 2002), we thus examined the expression of IL-25 in both the primary tumor and the metastatic lung. Whole breast tissues from MMTV-PyMT and wild-type (WT) control mice were extracted and analyzed for IL-25 expression. The mRNA analysis showed a significant increase of IL-25 expression in the tumor tissues compared with normal mammary tissues (Fig. 1C). Consistent with our findings in human breast tissues, the immunohistochemical analysis also revealed that tumor-infiltrating cells in MMTV-PyMT mice expressed high levels of IL-25 (Fig. 1B). To determine if IL-25 is expressed at the metastatic site, metastatic lung sections from tumor-bearing MMTV-PyMT mice were stained with IL-25 by IHC, but there was no IL-25 expression in both tumor and paratumor area (Fig. 1B), suggesting that IL-25 is mainly derived from the primary tumor sites.

Macrophages and T cells are often present at abundant numbers in various tumors (Mantovani et al., 2008). To determine whether these cells express IL-25 in the tumor microenvironment, immunofluorescence staining was performed in which macrophages were stained by anti-F4/80 whereas CD4+ T cells were stained by anti-CD4. Most F4/80+ or CD4+ cells were co-stained with anti-IL-25, suggesting that these cells are the major source of IL-25 in the tumor (Fig. 1D). For further confirmation, CD4+ T cells and CD11b+F4/80+Gr-1- macrophages were purified from tumor tissues, and analyzed for the mRNA expression of IL-25. Consistent with the immunofluorescence results, IL-25 expression in tumor-associated CD4+ cells and macrophages was >50 folds higher than in CD45- cells (Fig. 1E). These mRNA and IHC data also suggest that tumor cells are not a major source of IL-25.

### Anti-IL-25 treatment reduced lung metastasis

**Figure 2:**
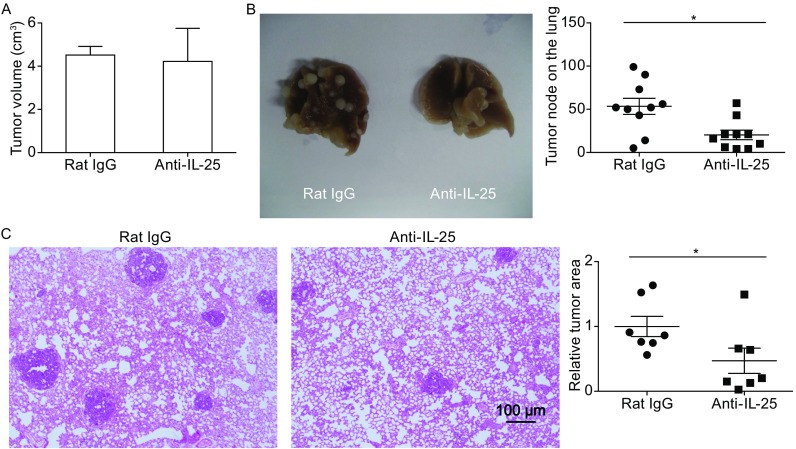
**Anti-IL-25 blocking antibody reduced lung metastasis**. The MMTV-PyMT mice were treated with either anti-IL-25 antibody or isotype control antibody starting from 4–5 weeks old, and sacrificed at 14 weeks old. (A) The size of primary tumors (mean ± SE). (B) Representative images of lung tissues isolated from the MMTV-PyMT and WT mice. The numbers of tumor nodes on the surface of lungs were shown as mean ± SE. (C) Representative H&E staining images of the lung tissues isolated from the MMTV-PyMT mice treated with either Rat IgG or anti-IL-25 antibody (×10 magnifications). The relative tumor areas were shown as mean ± SE and analyzed by Student’s *t* test

To address the function of IL-25 in tumor progression and metastasis, MMTV-PyMT mice were treated with an anti-IL-25 antibody or control antibody (Rat IgG) at the onset time of primary tumors (~4–5 week of age). The rat anti-mouse IL-25-specific blocking antibody was previously generated in our lab (Angkasekwinai et al., 2007), and widely used by the allergy field (Duan et al., 2010; Kaiko et al., 2010; Siegle et al., 2011; Angkasekwinai et al., 2013; Hong et al., 2014). The treatment continued for 10 weeks (200 μg, twice per week). Although the development of primary tumor (Fig. 2A) and the survival rate (Fig. S2) of MMTV-PyMT mice were not altered, anti-IL-25 antibody treatment reduced lung metastasis by >60%, as determined by the numbers of tumor nodes (Fig. 2B). In addition, the size of metastatic tumors was also significantly reduced as determined by tumor area inside the lung (Fig. 2C).

### CD4+ T cells in the tumor microenvironment expressed IL-17RB

**Figure 3:**
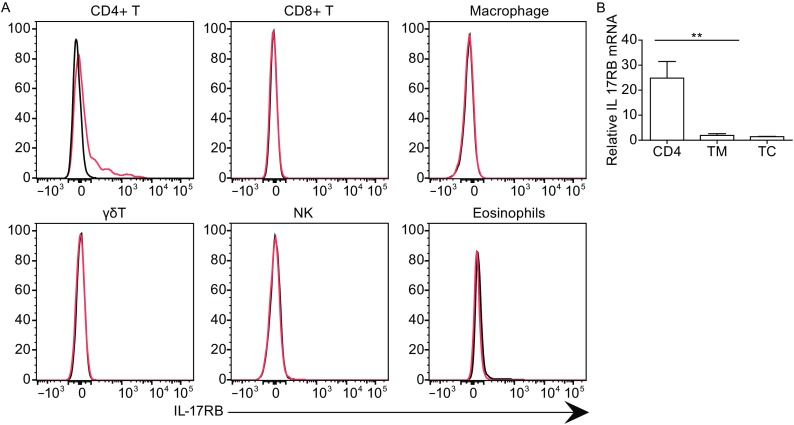
**Expression of IL-17RB**. The expression of IL-17RB was analyzed in mammary tumor-infiltrating cells isolated form the MMTV-PyMT primary tumor by flow cytometry (red) compared with isotype control (black) (A), or in sorted tumor infiltrating CD4 + (CD4:CD45+CD11b-CD3+CD4+) cells, macrophages (TM: CD45+CD11b+ F4/80+Gr-1-) and CD45- cells (TC) by relative mRNA expression (B)

IL-25 can act on both non-hematopoietic cells, for example breast epithelial cells, and hematopoietic cells, such as eosinophils, CD4+ T cells, NKT cells and type 2 innate lymphocytes etc. To determine the cells responding to IL-25 in the tumor microenvironment, we analyzed the expression of IL-17RB, the specific receptor for IL-25, in tumors (CD45-) and tumor-infiltrating immune cells, including CD4+ T cells (CD45+CD3+CD11b-CD4+), CD8+ T cells (CD45+CD3+CD11b-CD8+), NK cells (CD45+CD11b-DX5+), γδ T cells (CD45+CD3+CD11b-γδTCR+) and macrophages (CD45+CD3-CD11b+F4/80+Gr-1-), by flow cytometry (Fig. 3A). Interestingly, IL-17RB was selectively expressed by intra-tumor CD4+ T cells, but not CD8+ T, NK, γδ T cells or macrophages. For further confirmation, we sorted tumor macrophages (CD45+CD11b+F4/80+Gr-1-), tumor-infiltrating CD4+ (CD45+CD4+CD11b-), and CD45- cells, and then examined the mRNA level of IL-17RB in these cells. The mRNA results were consistent with the staining data that only CD4+ T cells expressed IL-17RB (Fig. 3B). In addition, we failed to detect IL-17RB in CD45- cells as determined by both surface staining and mRNA analysis, indicating that tumor cells may not respond to IL-25 directly. This is in contrast to the former report that IL-25 can cause apoptosis of breast cancer cells in vitro.

### Anti-IL-25 dampened Th2 response in the primary tumor tissue

**Figure 4:**
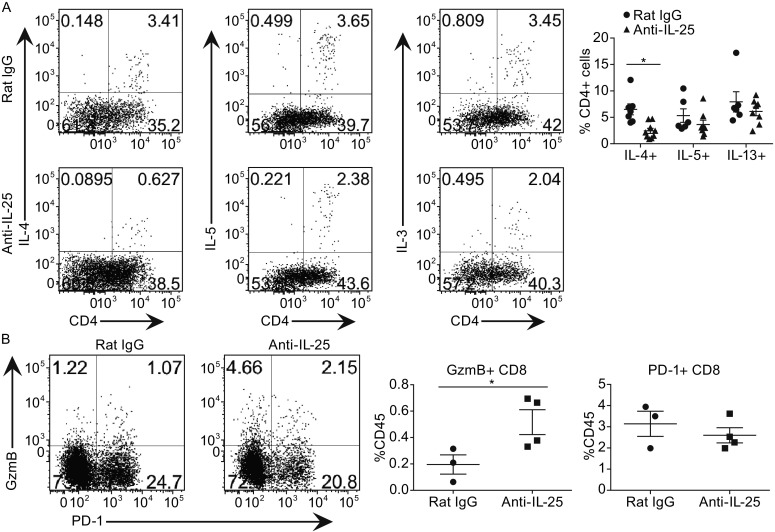
**Anti-IL-25 antibody treatment dampened Th2-responses in the primary tumor microenvironment**. The MMTV-PyMT mice were treated with either anti-IL-25 antibody or isotype control antibody starting from 4–5 weeks old, and sacrificed at 14 weeks old for the following analysis. (A) Expression of IL-4, IL-5 and IL-13 in tumor infiltrating cells (gated on CD4+ cells), and the statistic data were shown on the left. (B) Expression of PD-1 and Granzyme B in tumor infiltrating cells (gated on CD8+ cells), and the statistical data were shown on the left

IL-25 has been shown to promote Th2-associated pathology by inducing IL-4, IL-5, IL-13 and IL-9 expression (Fort et al., 2001; Angkasekwinai et al., 2007; Angkasekwinai et al., 2010). To address the possibility that IL-25 may also affect Th2 response inside primary breast tumors, we analyzed tumor-infiltrating IL-4-, IL-5- and IL-13-secreting CD4+ T cells in primary adenocarcinomas of MMTV-PyMT mice, and found that IL-25 blocking antibody significantly decreased the numbers of IL-4-secreting CD4+ cells while had no effect on IL-5 and IL-13 expression (Fig. 4A). We also analyzed ILC2, eosinophils, Th17, Treg, NK, γδ T cells, macrophages and MDSCs (MDSCs Myeloid-derived suppressor cells) (Fig. S3), since these immune cells have been shown to function in different tumor models. Interestingly, we did not observed any noticeable changes in the percentages and numbers of these cell types, except for CD8+ T cells, particularly Granzyme B+ CD8+ T cells, which were increased after anti-IL25 treatment (Fig. 4B).

### Anti-IL-25 treatment altered intra-tumor macrophage polarization

**Figure 5:**
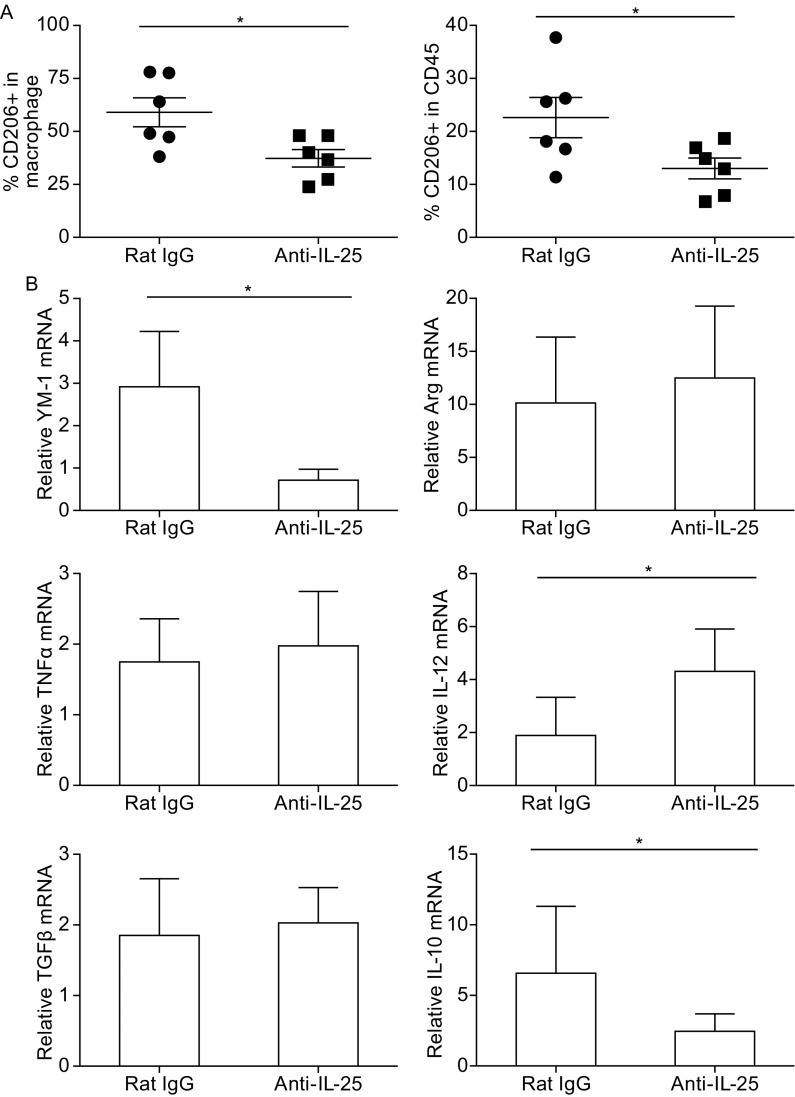
**Anti-IL-25 antibody treatment dampened the polarization of M2 macrophages in primary tumor**. The MMTV-PyMT mice were treated with either Rat IgG or anti-IL-25 antibody starting from 4 weeks old to 14 weeks old (3–7 mice/group), and then sacrificed at 14 weeks old for the following analysis. (A) Staining of CD206 positive macrophage populations in mammary carcinomas (shown as mean ± SE). (B) The relative mRNA level of YM-1 and Arg in sorted tumor macrophages or the relative mRNA level of *Il10, Il12, Tgfb* and *Tnfa* genes in the total tumor tissue

IL-4 can directly regulate the phenotypes and effector function of tumor-associated CD11b+Gr-1-F4/80+ macrophages, which were reported to promote the invasive and metastatic behavior of malignant mammary epithelial cells by secreting EGF and activating EGFR signaling programs in tumor epithelial cells (DeNardo et al., 2009). Since anti-IL-25 treatment resulted in a reduction of IL-4-producing Th2 cells, we then examined the polarization of tumor macrophages in the MMTV-PyMT tumor model. Interestingly, in the primary tumor, CD206+ macrophages were significantly reduced after anti-IL-25 antibody treatment (Fig. 5A), which were reported to promote tumor growth and metastasis (Van Dyken and Locksley, 2013). Consistently, the tumor tissue showed reduced expression of *Il10*, an M2 macrophage-associated gene, and increased expression of *Il12*, a marker gene for the M1 macrophages. In addition, Ym-1, a chitinase-like molecule expressed by alternatively activated tissue macrophages that can inhibit the function of effector CD8+ T cells, was also significantly reduced after anti-IL-25 antibody treatment (Fig. 5B). These results indicate that anti-IL-25 treatment reduced M2 macrophages in breast cancer.

## DISCUSSION

The understanding of cancer-associated inflammation in the tumor microenvironments would facilitate the development of novel immunotherapeutic approaches against various tumors by either stimulating anti-tumor or inhibiting pro-tumor inflammatory responses (Balkwill and Mantovani, 2012). Previous studies have established an important role of type 2 immune cells in promoting tumor progression and metastasis in breast cancer. Through targeting IL-25, an IL-17 family cytokine that positively regulates the initiation of type 2 immune response, we found that anti-IL-25 blocking antibody treatment led to more than 50% reduction of pulmonary metastasis in a spontaneous breast tumor model—the MMTV-PyMT transgenic mice, accompanied with reduced Th2 and M2 cells in the primary tumor tissue.

IL-25 was abundantly expressed in the primary sites of both human and mouse breast tumors, but not in the metastatic lung in the MMTV-PyMT tumor model. We found that only tumor-infiltrating CD4+ T cells, but not CD8+ T, macrophages or tumor cells express IL-17RB – the receptor for IL-25 (Fig. 3). In line with this evidence, treatment with anti-IL-25 blocking antibody significantly reduced IL-4-producing CD4+ Th2 cells in the MMTV-PyMT breast tumor model (Fig. 4A). Previous studies showed that IL-4 could enhance the invasive behavior of malignant MECs (mammary epithelial cells) and promote their dissemination and outgrowth in the lung through regulating the polarization of M2 macrophages. (DeNardo et al., 2009). Consistently, anti-IL-25 blocking antibody treatment also caused a decrease of M2 macrophages in the tumor microenvironment (Fig. 5) and noticeable increase of the Granzym B+ CD8+ T tumor killing cells (Fig. 4B), accompanied with reduced expression of *Il10* and increased expression of *Il12*. Interestingly, the therapeutic effect of anti-IL-25 antibody closely resembles that of anti-IL-4 blocking antibody as previously reported (DeNardo et al., 2009), in that both treatments only reduced pulmonary metastasis, but did not affect the primary tumor as well as the survival of tumor-bearing mice in the MMTV-PyMT breast tumor model. These results together highlight an essential role of the IL-25/Th2 axis in controlling tumor progression and tumor metastasis in breast cancer, suggesting that it may serve as a novel target for treatment of metastatic breast cancer.

IL-25 can be expressed by normal human breast epithelial cells (Furuta et al., 2011), and its level was found to be further elevated in human breast cancer biopsies in previous studies (Mombelli et al., 2015). In our study, we found that IL-25 was abundantly expressed in all four major types of human breast cancer patients, and both tumor cells and tumor-infiltrating cells expressed clearly high levels of IL-25. These findings indicate a role of IL-25 in breast tumor development. Indeed, two recent studies suggested an anti-tumor activity based on in vitro culture system and xenograft tumor model,through direct pro-apoptotic effect on tumor cells (Furuta et al., 2011) or indirect mechanism (Benatar et al., 2010), respectively. These reports are opposite to our observations that anti-IL-25 treatment only reduced lung metastasis, but did not affect the progression of primary tumors. Unlike the previous in vitro studies, we noticed that the tumor cells in the MMTV-PyMT tumor model did not express IL-17RB – the receptor for IL-25 (Fig. 3B), and thus could not respond directly to IL-25 treatment. Additionally, it has been suggested that only a minority population of breast cancer patients showed IL-17RB expression in the tumor cells. For examples, Furuta et al found that 13 out of 69 (18.8%) clinic sections from human breast tumors showed IL-17RB expression (Furuta et al., 2011), whereas Huang et al found that 38 out of 179 (21%, based on data presented in Fig. 6B in the paper) showed membranous staining of IL-17RB in the tumor cells (Huang et al., 2014). In line with the clinic data, the expression of IL-17RB is also variable among different breast tumor cell lines (Mombelli et al., 2015). Moreover, Mombelli et al failed to reproduce the anti-tumor activity of IL-25 on MCF-7, T47D or MDA-MB468 breast tumor cell lines, even they express IL-17RB (Mombelli et al., 2015). These contrasting data together highlights the complexity and heterogeneous nature of breast tumors, and thus IL-25 may play a distinct role in different tumor models. Nevertheless, our findings are consistent with the well-established role of IL-25 in boosting type 2 immune response and thereby enhancing tumor metastasis in breast tumor models.

IL-25 has been shown to be able to directly act on M2 macrophages and induce production of type 2 cytokines in different disease models (Cao et al., 2011; Rizzo et al., 2012). However, our studies showed that tumor macrophages in the MMTV-PyMT tumor model did not express IL-17RB and thus cannot directly respond to IL-25, similar to the alternatively activated macrophages as reported (Stolfi et al., 2011). Further studies suggest that these macrophages can be categorized into two groups: tumor-resident macrophages (CD206+YM-1+F4/80+Gr1-) and tumor-associated macrophages (CD206-YM-1- F4/80+Gr1-), with the former as the major tumor macrophage population that respond to anti-IL-25 antibody treatment. The result differs from the study using MMTV-PyMT mice on the C57/BL6 background, in which most tumor macrophages were characterized as CD206- tumor associated macrophages and were not affected by IL-4 deficiency (Franklin et al., 2014).

IL-25 can promote the expression of type 2 cytokines including IL-4, IL-5 and IL-13 in different in vitro and in vivo models. In this study, we noticed that anti-IL-25 treatment only reduced the IL-4-producing Th2 cells in the tumor microenvironment, but did not affect the expression of IL-5 and IL-13. Interestingly, a recent paper showed that IL-25 induced only IL-4 expression in Th2 cells at low dose (100 ng/ml), whereas induced IL-4, IL-5 and IL-13 expression at high dose (1000ng/ml), suggesting that IL-25 regulates the expression of different type 2 cytokine in a context-dependent manner (Swaidani et al., 2011).

Despite anti-IL-25 treatment significantly reduced lung metastasis in the MMTV-PyMT tumor model, it did not affect the growth of primary tumor and the survival of tumor mice. In this model, the primary tumor grew enormously huge and significantly hindered the moving activity of tumor-bearing mice when approaching death, whereas at the same time the lung metastasis still remains at minimal sizes. Our findings are consistent with several previous reports that inhibiting metastasis alone in the MMTV-PyMT tumor model could not improve the survival rate (Lin et al., 2001; DeNardo et al., 2009; Zabuawala et al., 2010), suggesting that death in this tumor model are mainly caused by uncontrolled growth of primary tumors. Therefore, anti-IL-25 did not affect the survival of tumor-bearing mice in our hands.

In summary, our study, for the first time, characterized tumor-infiltrating tumor macrophages as the major source of IL-25 in the MMTV-PyMT tumor mice, and demonstrated a critical role of IL-25 in promoting tumor metastasis through modulating type 2 immune response *via* targeting Th2 cells in breast tumor model. In addition, we found that IL-25 was broadly expressed in all the four major types of human breast cancer patients. Considering that death in human breast cancer patients are usually caused by tumor metastasis, a combination of tumor surgery with anti-IL-25 treatment may therefore provide a novel useful approach in treatment of human breast cancer.

## MATERIALS AND METHODS

### Mice and tumor sections

The MMTV-PyMT transgenic mice (FVB background) were purchased from Jackson laboratory. The mice were housed and bred under SPF condition in the animal facility at Tsinghua University. These experiments were approved by the Institutional Animal Care and Use Committee of Tsinghua University.

A total of 40 breast tumor tissue sections were obtained from the First Hospital of Jilin University, which include 10 of each major breast cancer intrinsic subtypes (Luminal A, Luminal B, Triple negative/basal-like, and HER2-enriched) (Sorlie et al., 2001; Prat et al., 2015). Written informed consent was obtained from all patients and the experimental protocol was approved by the Institutional Review Committee of Jilin University and the ethics committee of the First Hospital of Jilin University.

### Immunohistochemical and immunofluorescence staining

For immunofluorescence, frozen tissue sections were fixed in cold methanol for 10 minutes and air-dried. The sections were blocked for 30min with donkey serum and then incubated with a polyclonal goat anti-mouse IL-25 antibody (Santa Cruz, sc-22148) overnight, washed in PBS, and then incubated with TRITC-conjugated donkey anti-goat immunoglobulin and with anti-mouse F4/80-AF488 or anti-mouse CD4-AF488 for half an hour. The paraffin tissue sections from human and mouse primary tumor or lung were incubated with anti-IL-25 antibody (Millipore Cat. # 06-1080, which can bind both human and mouse IL-25) followed by visualization using rabbit Specific HRP/DAB Detection IHC Kit (ZSGB-BIO, PV-6001, ZLI-9017) (IHC: Immunohistochemistry) according to the manufacturer’s instructions. Staining was visualized by Zeiss microscope and analyzed by Image-Pro plus software.

### Anti-IL-25 administration and estimation of tumor growth and metastases

MMTV-PyMT mice were treated intraperitoneally with the anti-IL-25 antibody or control antibody (Rat IgG) around the onset time of primary tumors (~4–5 weeks’ old). The treatment continued for about ten weeks (200 μg per injection in 200 μl PBS, twice per week). The size of primary mammary tumors was measured using electronic calipers and calculated as (length × width × height)/2, pulmonary tissue sections were stained with H&E for analysis of metastatic tumors by light microscope and Image-Pro Plus. In this study, the IL-25 blocking antibody (clone# 35B) was initially generated and characterized by our lab through screening a panel of rat anti-mouse IL-25 IgG for the ability to inhibit the binding of IL-25 to 293 cells transfected with IL-17RB (Angkasekwinai et al., 2007). This antibody is now commercially available at BioLegend (Cat#514404)

### Real-time RT-PCR

Total RNA was extracted from mouse breast tissue or sorted cells by Trizol reagent (Invitrogen) according to manufacturer’s instructions. Two micrograms of total RNA was reversely transcripted into cDNA by using cDNA Reverse Transcription Kit (Invitrogen). Real-time qRT-PCR was performed in CFX96 Touch™ Real-Time PCR Detection System (BioRad) using iTaq™ Universal SYBR® Green Supermix (BioRad) and specific primers for *Il25*, *Il17rb*, *Arg*, *Cd206*, *Ym1, Il10,Il12, Tgfb and Tnfa*, and normalized to the housekeeping gene GAPDH (Supplementary data 4).

### Preparation of tumor-infiltrating cells

Tumor tissues from MMTV-PyMT mice were minced and digested in DMEM medium containing 1 mg/mL collagenase A, 2.0 units/ml DNase (all from Roche) for 2 h at 37°C with periodic vortex. Tumor infiltrating cells were purified by Percoll density gradient centrifugation, stained with different surface markers as indicated in the paper, and then analyzed by flow cytometry.

### FACS analysis and cell sorting

Fluorochrome labeled anti-mouse mAbs specific for CD45(30-F11), CD45R (RA3-6B2),CD3 (17A2), CD11b (M1/70), CD4 (GK1.5), CD8 (53-6.7), CD49b(DX5), γδTCR(GL-3),MHC class II (M5/114.15.2), F4/80 (BM8), CD25 (PC61), Ly6G (1A8), Ly6C (AL-21), Ly- 6C/G (RB6/8C5), Siglec-F (E50-2440), ST2 (DIH9), and IL-17RB antibody (752101) were used for flow cytometry analyses. For intracellular staining, purified cells were stimulated with PMA (50 ng/ mL)/ionomycin (500 ng/mL) (Sigma-Aldrich) with GolgiStop (BD Pharmingen, NJ) for 4 hrs and stained with fluorochrome-labeled anti-mouse mAbs specific for CD206 (C068C2),GzmB (NGZB) Foxp3 (MF23), IL-4 (BVD4- 1D11), IL-5 (TRFK5), IL-13 (eBio13A) (BD Pharmingen/BioLegend, San Diego, CA/eBiosciences, San Diego, CA). Cells were analyzed with BD LSRFortessa™ cell analyzer (BD Biosciences, San Jose, CA) and the analysis was conducted with FlowJo software. Macrophages, CD4+ T cells and CD45 negative tumor cells were sorted using a FACSAria (BD) according to their corresponding surface markers.

### Statistical analysis

The statistic significance of our data was analyzed by Student’s *t* test using Prism (version 5.00; GraphPad Software). (*P < 0.05; **P < 0.01; ***P < 0.001). All the experiments in the paper was repeated 2–4 times with consistent results.

## Electronic supplementary material

Below is the link to the electronic supplementary material.

Supplementary material 1 (PDF 533 kb)
