# Supplementary material for: IL-25 blockade inhibits metastasis in breast cancer
Source: Protein Cell. 2016 Dec 1;8(3):191–201. doi: 10.1007/s13238-016-0345-7 (PMC5326622; doi:10.1007/s13238-016-0345-7)
Supplement: Supplementary file 1 — Supplementary material 1 (PDF 533 kb) [file 13238_2016_345_MOESM1_ESM.pdf]

A

B

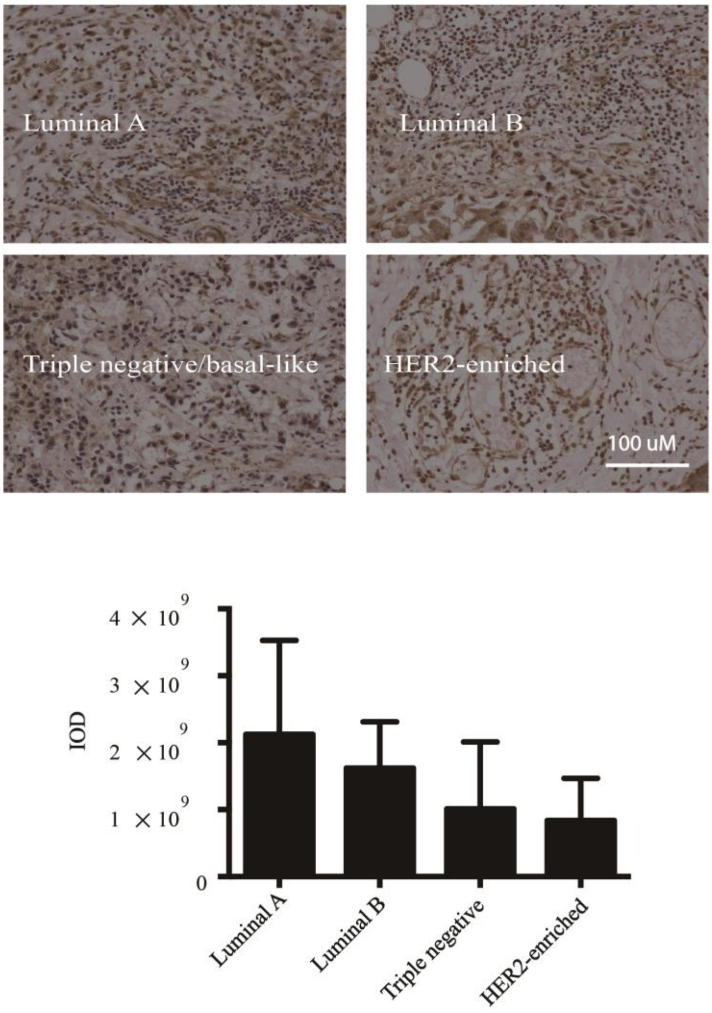

Supplementary figure 1 . Expression of IL-25 in different types of human breast tissues.
Paraffin-embedded human breast cancer sections of four types including Luminal A (ER+ and/or PR+, HER2-, low Ki67), Luminal B (ER+ and/or PR+, HER2+ or HER2- with high Ki67), Triple negative/basal-like (ER-, PR-, HER2-), and HER2-enriched type (ER-, PR-, HER2+) were analyzed by immunohistochemistry after staining with anti-IL-25 mAb (40x magnification). (A).The data from a presentative of ten experiments are shown. (B). IL-25+ cells in four groups were characterized through counting integrated optic density by Image Pro Plus. There was no difference among four groups. ER, Estrogen receptor. PR, progesterone receptor.

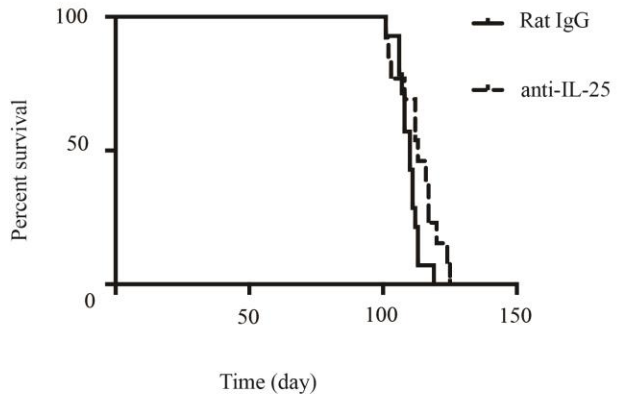

Supplementary figure 2. Anti-IL-25 antibody did not affect the survival of the MMTV-PyMT mice.

MMTV-PyMT mice of 4-5 weeks old were treated with either anti-IL-25 antibody or isotype control antibody. The treatment continued for about ten weeks with 200ug per injection in 200ul PBS, twice per week. Survival curves were compared using GraphPad Prism Software. There was no significant difference between Rat IgG group and anti-IL-25 group.

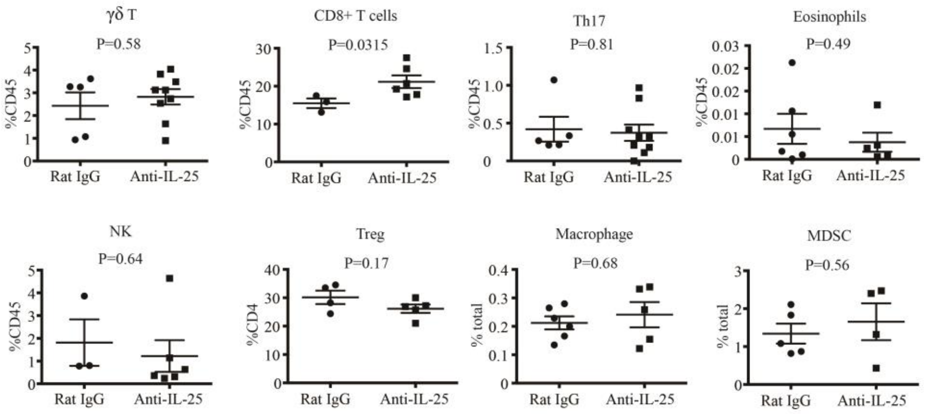

Supplementary figure 3. The change of tumor infiltrating immune cells after anti-IL-25 treatment.
MMTV-PyMT mice of 4-5 weeks old were treated with either anti-IL-25 antibody or isotype control antibody. The treatment continued for about ten weeks with 200ug per injection in 200ul PBS, twice per week. When the mice were 14 weeks old, tumor infiltrating immune cells were calculated and compared using GraphPad Prism Software. All mice in each group were calculated as shown, and one dot represents one mouse. Except for CD8+ T cells, there was no significant difference between Rat IgG group and anti-IL-25 group.

Supplementary Table 1 Primers for Q-PCR

Primer	Forward	Reverse
IL-25	5'-TTGGAGCTATGAGTTGGACAGGGA-3'	5'-AGACCGTCTGGTTGTGGTAAAGTG-3'
IL17RB	5'-TGTGTTGGACCATCCACTCT-3'	5'-AGTGTTGCTGATCTTGGCTG-3'
Arg	5'-TTTTTCCAGCAGACCAGCTT-3'	5'-CATGAGCTCCAAGCCAAAGT-3'
CD206	5'-CAGGTGTGGGCTCAGGTAGT-3'	5'-TGTGGTGAGCTGAAAGGTGA-3'
YM-1	5'-TTTCTCCAGTGTAGCCATCCTT-3'	5'-TCTGGGTACAAGATCCCTGAA-3'
NOS	5'-TCCAGGGATTCTGGAACATT-3'	5'-GAAGAAAACCCCTTGTGCTG-3'
TGF	5'-AAGTTGGCATGGTAGCCCTT-3'	5'-GCCCTGGATACCAACTATTGC-3'
IL-1	5'-AGGTCAAAGGTTTGGAAGCA-3'	5'-TGAAGCAGCTATGGCAACTG-3'
GAPDH	5'-CTCGGCCTTGACTGTGCCGT-3'	5'-AGTGCCAGCCTCGTCCCGTA-3'
